# A Cross-Sectional Exploratory Study of Cardiovascular Risk Biomarkers in Non-Obese Women with and without Polycystic Ovary Syndrome: Association with Vitamin D

**DOI:** 10.3390/ijms25126330

**Published:** 2024-06-07

**Authors:** Manjula Nandakumar, Priya Das, Thozhukat Sathyapalan, Alexandra E. Butler, Stephen L. Atkin

**Affiliations:** 1 Royal College of Surgeons of Ireland, Adliya P.O. Box 15503, Bahrain; mnandakumar@rcsi.com (M.N.); pdas@rcsi.com (P.D.); satkin@rcsi.com (S.L.A.); 2 Academic Endocrinology, Diabetes and Metabolism, Hull York Medical School, Hull HU6 7RU, UK; thozhukat.sathyapalan@hyms.ac.uk

**Keywords:** polycystic ovary syndrome, PCOS, vitamin D, cardiovascular risk, biomarkers, 25 hydroxyvitamin D_3_, 1,25 dihydroxyvitamin D_3_

## Abstract

Vitamin D is proposed to have a protective effect against cardiovascular disease, though the mechanism is unclear. Vitamin D deficiency is common in polycystic ovary syndrome (PCOS), where it is strongly related to obesity, insulin resistance (IR) and risk of cardiovascular disease. To determine if the inherent pathophysiology of PCOS or vitamin D levels are linked to dysregulation of cardiovascular risk proteins (CVRPs), a study in non-obese women with PCOS and without IR was undertaken. Our hypothesis was that the levels of vitamin D_3_ and its active metabolite would be associated with CVRPs comparably in women with and without PCOS. In women with PCOS (*n* = 29) and controls (*n* = 29), 54 CVRPs were determined by Slow Off-rate Modified Aptamer (SOMA)-scan plasma protein measurement and correlated to 25-hydroxyvitamin D_3_ (25(OH)D_3_) and the active 1,25-dihydroxyvitamin D_3_ (1,25(OH)_2_D_3_) measured by gold standard isotope-dilution liquid chromatography tandem mass spectrometry. Women with PCOS had comparable IR and systemic inflammation (normal C-reactive protein) to control women, though had higher free androgen index and anti-Mullerian hormone levels. 25(OH)D_3_ and 1,25(OH)_2_D_3_ levels did not differ between groups. Nine CVRPs were higher in PCOS (*p* < 0.05) (Galectin-9, Brother of CDO, C-motif chemokine 3, Interleukin-18 receptor-1, Thrombopoietin, Interleukin-1 receptor antagonist protein, Programmed cell death 1 ligand-2, Low-affinity immunoglobulin gamma Fc-region receptor II-b and human growth hormone), whilst 45 CVRPs did not differ. 25(OH)D_3_ correlated with five CVRPs in PCOS and one in controls (*p* < 0.05). Despite the women with PCOS not exhibiting overt systemic inflammation, 9 of 54 CVRPs were elevated, all relating to inflammation, and 5 of these correlated with 25(OH)D_3,_ suggesting an ongoing underlying inflammatory process in PCOS even in the absence of obesity/IR.

## 1. Introduction

Polycystic ovary syndrome (PCOS) is reported to have a prevalence of 5–10% of women and is the most prevalent endocrine abnormality in premenopausal women [1]. PCOS is diagnosed on the basis of recognized international criteria after exclusion of other conditions [2], and it is estimated that 70% of women with PCOS remain undiagnosed [3]. PCOS is considered a metabolic disease with an increased prevalence of hypertension, dyslipidemia, type 2 diabetes and enhanced cardiovascular risk factors in affected women [4]. The pathophysiological features of PCOS are likely caused by oxidative stress, chronic inflammation, hyperandrogenemia, vitamin D insufficiency and insulin resistance (IR) [5,6], which result in dysregulation of cellular biomarkers such as heat shock proteins [7], complement proteins and coagulation markers [8]. These pathophysiological features are primarily driven by the combination of obesity and IR [9,10,11,12], though the exact underlying mechanisms remain unclear.

Obesity is common in PCOS with over 60% of women being overweight or obese; however, the prevalence of PCOS among non-obese populations suggests that it is not the only underlying factor in the pathogenesis of PCOS [13]. Obesity does, however, exacerbate the underlying pathophysiology, especially cardiovascular risk factors such as insulin resistance, glucose intolerance and dyslipidemia. This is highlighted by the fact that substantial weight loss markedly improves cardiovascular risk markers following bariatric surgery [14], but the limited data available do not indicate that medical interventions decrease cardiovascular events or delay the onset of diabetes [15]. PCOS is associated with IR, an increased risk of diabetes, non-alcoholic fatty liver disease (NAFLD) and increased cardiovascular (CV) risk, independently of obesity [16,17]. Chronic systemic inflammation is seen in obesity and in PCOS with high-sensitivity C-reactive protein (hs-CRP; a measure of systemic inflammation) being significantly increased in both conditions [18], and has been shown to be a strong predictor of cardiovascular events in women [19]. IR results from a defect in insulin action, including in insulin-mediated glucose transport and its signaling pathway [20].

**Figure 1:**
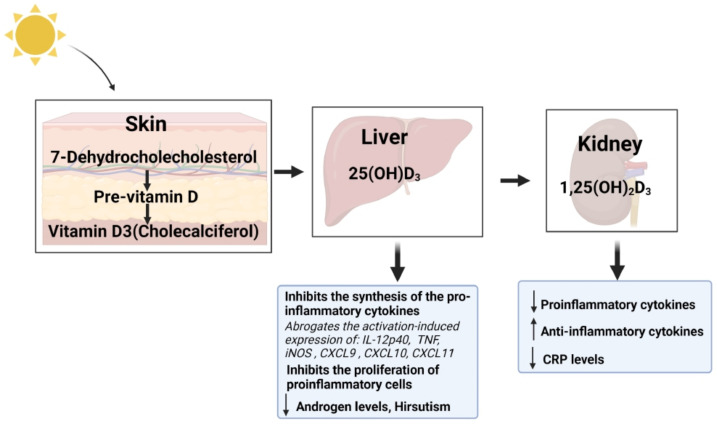
In the skin, 7-dehydrocholesterol is converted to previtamin D_3_ and is thermally isomerized to vitamin D_3_. Vitamin D_3_ from the skin is transported to the liver via Vitamin D-binding protein (DBP) that also transports 25 hydroxy vitamin D (25(OH)D_3_) to the kidney. In the kidney, 25(OH)D_3_ undergoes a second hydroxylation step via 1-alpha-hydroxylase Cytochrome P450 Family 27 Subfamily B Member 1 (Cyp27B1), thus converting to the active 1,25 (OH)_2_D_3_.

Vitamin D_3_, also known as cholecalciferol, is produced naturally in the skin upon exposure to ultraviolet B radiation (UV-B). This UV-B radiation acts upon 7-dehydrocholesterol, which is hydroxylated at position 25 to form 25-hydroxy vitamin D_3_ (25(OH)D_3_). 25(OH)D_3_ is transported to the kidney where 1-alpha hydroxylase converts it to the active form, 1,25(OH)_2_D_3_ [21] (Figure 1). Both renal and non-renal tissues have the ability to convert 25(OH)D_3_ to 1,25(OH)_2_D_3_. However, the process of conversion differs between these tissues with extra-renal tissues relying upon macrophages to produce 1,25(OH)_2_D_3_ through the type 2 interferon response [22]. 1,25(OH)_2_D_3_ binds to the vitamin D receptor (VDR) where it heterodimerizes with the retinoid X receptor [23], with a more rapid mechanism of action through binding to membrane VDR or through the 1,25D-membrane-associated, rapid-response steroid-binding protein receptor with activation of protein kinases A and C [24]. The bound 1,25(OH)_2_D_3_ and VDR element regulates the transcription of the insulin gene [25]. Vitamin D deficiency is associated with obesity, increased IR and elevated levels of testosterone, all of which are common in PCOS [26,27]; moreover, vitamin D replacement has been reported to be of benefit for IR and steroidogenesis in obese women with PCOS. Vitamin D deficiency is common in women with PCOS, and it has been reported that 67–85% exhibit severe deficiency [28,29]. The vitamin D deficiency reported to be associated with IR is affected by obesity [30] with vitamin D deficiency increasing with obesity as the vitamin D is sequestered in adipose tissue. Vitamin D has been reported to play significant roles in systemic inflammation, oxidative stress and mitochondrial function and is a potent antioxidant [31]. There is ongoing controversy regarding the anti-inflammatory properties of 25(OH)D_3_, with some studies reporting an effect, while others have shown none [32]. However, a study on vitamin D supplementation did demonstrate a decrease in C-reactive protein (CRP) levels and stabilization of the anti-inflammatory cytokine, interleukin 10, in obese individuals, indicating a potential anti-inflammatory effect [33]. Further, 1,25(OH)_2_D_3_ is reported to have inherent anti-inflammatory effects through alterations in macrophage and T cell responses [34,35,36] (Figure 1).

Obesity and IR are closely associated with PCOS, so that they are not easily accounted for statistically. Thus, only a study of non-obese PCOS women without IR could answer the question of whether the inherent pathophysiology of PCOS or vitamin D levels are related to differences in cardiovascular risk proteins (CVRPs). To address this, we assessed the association of vitamin D_3_ (25(OH)D_3_) and its active metabolite 1,25(OH)_2_D_3_ with CVRP levels in non-obese, non-IR women with PCOS compared to a matched non-PCOS control population. The hypothesis was that 25(OH)D_3_ and 1,25(OH)_2_D_3_ levels would correlate with CVRPs but that there would be no difference in CVRP levels between non-obese PCOS and control groups.

## 2. Results

Demographic, hormonal and metabolic data for the 29 PCOS subjects and 29 controls are shown in Table 1. The two cohorts were weight- and age-matched and had neither IR nor evidence of increased systemic inflammation (C-reactive protein was not elevated and did not differ between the groups), though subjects with PCOS did have hyperandrogenemia and elevated anti-Müllerian hormone (AMH). 25(OH)D_3_ and the active form 1,25(OH)_2_D_3_ did not differ between groups.

### 2.1. Cardiovascular Risk Protein Levels

Limma analysis revealed that 9 of the 54 CVRPs measured (Table 2) were elevated in PCOS compared to controls (Table 3), namely Galectin-9 (LEG9), Brother of CDO (BOC), C-motif chemokine 3 (MIP-1a), Interleukin-18 receptor 1 (IL-18 Ra), Thrombopoietin (Tpo), Interleukin-1 receptor antagonist protein (IL-1Ra), Programmed cell death 1 ligand 2 (PD-L2), Low-affinity immunoglobulin gamma Fc region receptor II-b (FCG2B) and human growth hormone (HGH).

### 2.2. Functional and Pathway Enrichment for Dysregulated Proteins

Functional enrichment of the upregulated proteins in the PCOS cohort generated gene ontology lists. Four proteins with GO:0006954 (inflammatory response), four with GO:0006955 (immune response) and three with GO:0032760 (positive regulation of tumor necrosis factor production) were significantly overrepresented in biological process (BP) terms (Table 4).

Five out of nine proteins were significantly enriched with Kyoto Encyclopedia of Genes and Genomes DAVID: Database for Annotation, Visualization and Integrated Discovery (KEGG)_Pathway term: cytokine–cytokine receptor interaction (Table 4).

### 2.3. Correlation Analyses between 25(OH)D_3_, 1,25(OH)_2_D_3_ and Cardiovascular Risk Proteins

There was a significant positive correlation between 25(OH)D_3_ and the active form 1,25(OH)_2_D_3_ (*p* = 0.01) in women with and without PCOS.

Among the PCOS group, Galectin-9 (LEG9) showed a strong positive correlation with 25(OH)D_3_ (r = 0.518, *p* = 0.019). The proteins that were negatively correlated to 25(OH)D_3_ in the PCOS cohort were alpha-L-iduronidase (IDUA) (r = −0.570, *p* = 0.009), angiopoietin (r = −0.500, *p* = 0.025), Dickkopf-related protein (DKK1) (r = −0.45, *p* = 0.04) and Gro-a (r = −0.437, *p* = 0.04). C-C motif chemokine 1 (TARC) was positively correlated, and Pappalysin-1 (PAPPA) was negatively correlated to 1,25(OH)_2_D_3_ (r = 0.68, *p* = 0.0009 and r = −0.47, *p* = 0.03) in the control group. Matrilysin (MMP-7) was positively correlated to 25(OH)D_3_ (r = 0.412, *p* = 0.02) in the control group (Table 5).

### 2.4. Protein–Protein Interactions

**Figure 2:**
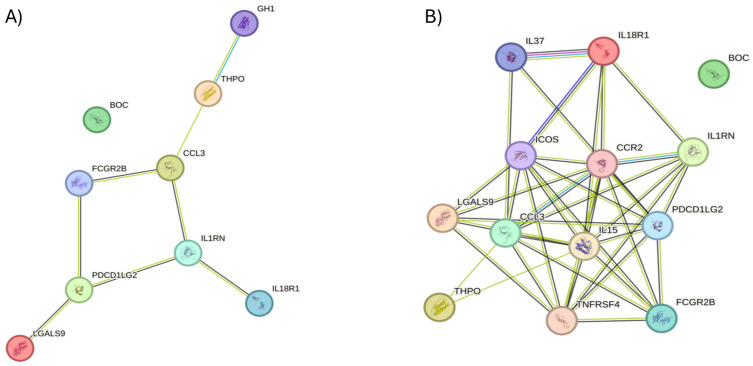
(**A**) The STRING version 12.0 interaction network was used to generate the protein–protein interaction between the dysregulated proteins identified in SOMAscan assay in non-obese women with PCOS versus controls; (**B**) Protein–protein interactions between the dysregulated proteins and the predicted immediate binding partners. ‘Co-expression’ is indicated by a black edge.

STRING 12.0 (Search Tool for the Retrieval of Interacting Genes) was used to visualize the known and predicted protein–protein interactions (PPIN) for proteins that were dysregulated in PCOS compared to controls, as identified by the SOMAscan assay (https://string-db.org/). These interactions between proteins are evidence-based and collated from databases, experiments, neighborhood, gene fusion, co-occurrence, text mining, co-expression and homology (Figure 2A). The majority of the dysregulated proteins in the PCOS cohort showed evidence of ‘coexpression’ in the PPIN analysis of STRING. The dysregulated proteins seem to have direct interactions with cytokines (interleukins) and immune cell-stimulating factors as per the STRING database (Figure 2B), indicating their involvement in inflammatory and immune responses.

### 2.5. Multivariable Regression and Predictive Value of the Variables

**Figure 3:**
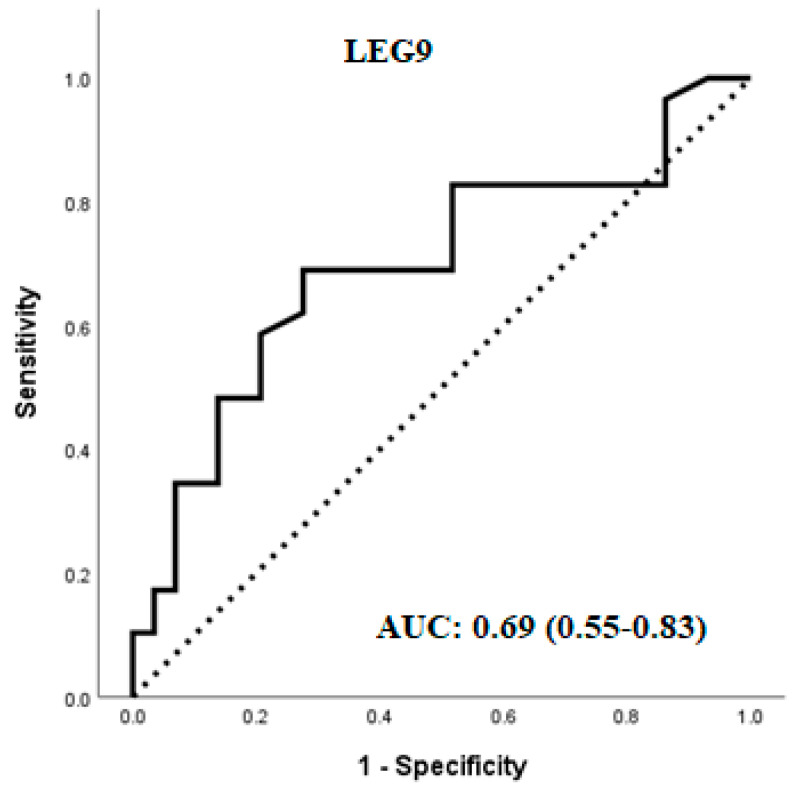
ROC curve of LEG9 expression level. The AUC indicates the diagnostic power of LEG9 in non-obese PCOS (0.69).

The dysregulated proteins were subjected to stepwise multivariable logistic regression to model their association with PCOS in non-obese subjects. Only LEG9 was included in the predictive model for discriminating PCOS in non-obese patients versus the control, with an area under the curve (AUC) of 0.69 (Figure 3). According to the receiver operating characteristic (ROC) curves and Youden’s Index, the optimal cutoff value of LEG9 was 968.7 relative fluorescent units (RFU), with 69.0% sensitivity and 72.4% specificity.

## 3. Discussion

This study was designed to answer the question of whether the inherent pathophysiology of PCOS or vitamin D levels are related to differences in CVRPs. Our hypothesis was that 25(OH)D_3_ and 1,25(OH)_2_D_3_ levels would correlate with CVRPs, but that CVRP levels would not differ between women with PCOS without obesity or IR and a control group. The non-obese cohort of PCOS patients recruited for this study were matched to controls for BMI, IR and CRP as well as having equivalent levels of 25(OH)D_3_ and 1,25(OH)_2_D_3_. However, our hypothesis was disproven as the results showed that there were nine dysregulated CVRPs in the PCOS cohort. As vitamin D levels were equivalent between cohorts, we also hypothesized that there would be no correlation differences between 25(OH)D_3_ or 1,25(OH)_2_D_3_ and the CVRPs. However, four CVRPs were found to correlate negatively and one positively with 25(OH)D_3_ in the PCOS cohort, whilst one CVRP correlated with 25(OH)D_3_ in the control cohort. Two CVRPs correlated with 1,25(OH)_2_D_3_ in the control cohort. The CVRPs that were elevated and those that were correlated to vitamin D in PCOS were associated with inflammation and the immune response, as evidenced by STRING protein–protein interactions; this would accord with the chronic low-level inflammation that may be involved in the pathogenesis of PCOS [37], and that is not influenced by the presence of excessive weight or obesity [38]. These results would suggest that the alterations in CVRPs are both an inherent feature of PCOS that are likely indicative of early inflammation as well as due to changes in vitamin D metabolites.

In total, nine dysregulated CVRPs were found in non-obese PCOS subjects compared to the controls, and functional enrichment and gene ontology analysis of these indicated the enhancement of GO terms associated with inflammatory and immune responses, pathways that are actively regulated in PCOS [39,40], as detailed below, and that may be fundamentally involved in the pathogenesis of PCOS.

LEG9 is significantly enriched in cytokine and interleukin-2 signaling and has been shown to play a key inflammatory role in rheumatoid arthritis (RA) and angiogenesis, resulting in the expansion of the vascular system and increased attachment and infiltration of white blood cells through ERK1/2 p38 and Jnk pathways [41]. In addition, LEG9 was shown to be involved in monocyte recruitment and atherosclerotic plaque progression [42]. This may be important in PCOS, as there is increased cardiovascular risk [4], particularly in obese, IR individuals, so it was surprising to see LEG9 being elevated in the absence of an associated increase in CRP, and this suggests that it may be a biomarker candidate in non-obese PCOS.

Elevation in macrophage inflammatory protein (MIP-1α) was also seen in PCOS, which accords with the increased levels of MIP-1α reported previously in women with PCOS [38]. MIP-1α/CCL3 is an inflammatory chemokine produced by cells during infection or inflammation. CCL3 activates the PI3K/AKT signaling pathway and enhances the expression level of pro-inflammatory cytokines such as IL-6, IL-1β and TNF-α in addition to upregulating CD4+T cells to mediate the inflammatory response [43]. MIP-1α-induced signaling involves activation of the AKT/protein kinase B (PKB) and the mitogen-activated protein kinase (MAPK) pathway [44], which are activated in PCOS. Though no difference in MCP1 levels in follicular fluid between PCOS and non-PCOS women was reported, circulating MCP1 was upregulated in PCOS [45].

Elevation in IL-18R was found in PCOS. IL-18R belongs to the IL-1R family, and the IL-18R complex is composed of the IL-18Rα and IL-18Rβ chains [46]. Both the IL-18Rα and IL-18Rβ chains are thought to be essential for IL-18-mediated signaling [47]. IL-18 shares properties similar to those of other proinflammatory cytokines, such as increases in chemokine production and cell adhesion molecules. It is also involved in production of IFN-γ, T cell polarization and induction of FAS ligand [48]. Soluble IL-18R complex showed an antagonistic effect in vivo; it plays an important role in the inflammatory process in allergic asthma [49] and is upregulated in obesity and insulin resistance [50]. IL-18 is increased in PCOS patients and correlates with insulin resistance, obesity and hyperandrogenism [51], suggesting that it, too, could serve as a potential biomarker in non-obese non-IR PCOS.

Thrombopoietin (TPO) was elevated in PCOS; TPO plays a central role in controlling the development of megakaryocytes and promoting the generation of platelets. Inflammatory conditions can increase the secretion of TPO. TPO acts by initiating intracellular signaling, including activation of the JAK2/signal transducers and activators of the transcription (STAT) pathway [52]. The results of a study on non-obese women with PCOS concluded that platelet dysfunction might play a significant role in the development of cardiovascular risk in women with PCOS, indicating the possible role of TPO in PCOS [53]. Thrombopoietin was earlier shown to be upregulated in obesity [54]; in our study, we also found upregulation of TPO in non-obese women with PCOS, indicating that its expression might be independent of weight/BMI.

Interleukin 1 receptor antagonist (IL-1Ra), elevated in non-obese PCOS, is a protein that binds to the IL-1 receptor and blocks the binding of both IL-1 alpha and -beta without inducing its signaling pathway [55]. IL-1Ra is a critical mediator of inflammatory processes and is speculated to play a role in the pathogenesis of PCOS. A study of the association of IL-1Ra gene polymorphisms with PCOS and related metabolic conditions showed that allele II in intron 2 of IL-1Ra had a strong association with several metabolic characteristics associated with PCOS [56]. Elevated levels of IL-1Ra in women with PCOS have been primarily attributed to obesity; elevated serum IL-1Ra levels were found to be a predictive factor for 2 h glucose levels, irrespective of BMI, indicating a potential association between increased IL-1Ra and impaired glucose metabolism [57].

Programmed death ligands, namely PD-L1 and PD-L2, are negative co-stimulatory molecules that control T cell motility and formation of an immune synapse between T cells and antigen-presenting cells (APCs). The upregulation of PD-L2 may modulate the chronic inflammation of colonic mucosa in inflammatory bowel disease [58], and its increased expression in sepsis patients indicates potential involvement in the septic pathogenesis that occurs with systemic immune activation, leading to multiple organ dysfunction [59]. In a study of single-nucleotide polymorphisms (SNPs) in PCOS, both PD-1 and PD-L1 SNPs were suggested to be related to the pathogenesis of PCOS.

Low-affinity immunoglobulin gamma Fc region receptor II-b (FCGR2B/FcγRIIB) plays a significant role in governing various aspects of immune and inflammatory responses. FcγRIIB is specifically involved in the intricate regulation of the immune system against infections [60]. In macrophages, engagement of FcγRIIB can reduce FcγR-mediated phagocytosis and cytokine release (including tumor necrosis factor (TNF), interleukin-6 (IL-6), IL-1α and neutrophil chemoattractants), as well as Toll-like receptor 4 (TLR4)-mediated activation. FcγR-mediated phagocytosis and cytokine release and Toll-like receptor-mediated activation are balanced by FcγRIIB [61,62]. Inhibitory signaling has been shown in all cell types that express FcγRIIB except for follicular dendritic cells (FDC), which express high levels of FcγRIIB in germinal centers where it is required for FDC activation [63]. Its role in PCOS is unclear as this is the first report of it being elevated in this condition.

Human growth hormone (HGH) was found to be higher in PCOS than controls, in accord with a study that showed that women of normal weight with PCOS and hyperinsulinemia presented with high HGH levels in response to the l-dopa test, suggesting that the action of GH and insulin-like growth factor-1 (IGF-1) might be responsible for the elevation in luteinizing hormone (LH) and the consequent hyperandrogenic anovulation observed in normal weight women with PCOS [64], but our results suggest that IR may not need to be present for HGH elevation.

Brother of CDO (BOC) was increased in PCOS; it encodes a member of the immunoglobulin/fibronectin type III repeat family. It is a component of a cell-surface receptor complex that mediates cell–cell interactions between muscle precursor cells and promotes myogenic differentiation. Its role in PCOS is unclear as this is the first report of it being elevated in this condition.

This study sheds light on the fact that when obesity, insulin resistance and overt systemic inflammation are accounted for, there are still underlying subtle systemic increases in the inflammatory cardiovascular proteins. Studies have shown that 75% of non-obese women have IR [65], and, potentially, the findings here could be exaggerated in the presence of IR, studies that need to be done. However, these subtle changes may need to be considered with potential long-term monitoring, as increasing weight and IR could amplify these proteins, and overt systemic inflammation may then supervene. Given that the CRP, vitamin D and its metabolite levels were not different among the control and PCOS cohorts in this study, it is suggestive that the subclinical inflammation seen (as evidenced by overexpression of CVRPs) may be due to the inherent underlying pathophysiology of PCOS, and with increasing weight above the normal range (body mass index > 25 kg/m^2^) this process may be exacerbated. Binding of vitamin D metabolites to the vitamin D receptor (VDR) is essential for transcription of the insulin gene; therefore, vitamin D deficiency could play a role in the development of IR. In addition, several studies have reported the production of excess pro-inflammatory mediators in PCOS subsequently leading to IR, obesity and CVD [66]. Vitamin D supplementation has shown a significant role in improving IR and obesity, thus preventing CVD. The chronic inflammation in PCOS and low vitamin D levels are likely both risk factors for the development of CVD [67,68].

Only one CVRP (MMP7) correlated to 25(OH)D_3_ in the control cohort, and this correlation was lost in the PCOS cohort. MMP7 is an endopeptidase involved in breakdown of the extracellular matrix and thereby plays a role in wound healing and tissue remodeling. Any dysregulation in MMPs is associated with scarring and fibrosis in inflammation and wound healing. A study investigating the transcriptome profile of endometrium in PCOS showed significantly lower expression of MMP7 in PCOS compared to healthy controls [69]. The inflammatory environment inherent to PCOS could potentially cause loss of the association of MMP7 with 25(OH)D_3_ in these non-obese PCOS subjects.

A negative correlation of angiopoietin was observed with 25(OH)D_3_ in the PCOS cohort. There are contradicting reports on levels of angiopoietin in PCOS women. Tal et al. reported higher angiopoietin-2 levels in follicular fluid following ovarian stimulation in PCOS versus non-PCOS women [70]; conversely, Mariana et al. reported no changes in angiopoietin-2 levels in the follicular fluids in PCOS versus non-PCOS women [71].

DKK-1 negatively correlated with 25(OH)D3 in the PCOS cohort in our study. DKK1 has been reported to be significantly associated with PCOS [72]. A negative correlation of IDUA with 25(OH)D_3_ was found in the PCOS cohort. IDUA is required for lysosomal degradation of glycosaminoglycans (GAG), and GAG levels are higher in follicular fluid of PCOS women [73]. A negative correlation of Chemokine (C-X-C motif) ligand 1 (CXCL1; also known as Gro-a) with 25(OH)D_3_ was also found in the PCOS cohort. CXCL1 is a mediator of neutrophil activity and is involved in the processes of angiogenesis, inflammation, wound healing and tumorigenesis [74]. Whilst it was not upregulated in PCOS in this study, it is reported to be associated with PCOS. CXCL1 is inversely associated with sex hormones in PCOS [75]. CXCL1 gene expression trended towards a positive association with BMI in PCOS [76] and is associated with obesity [77], suggesting that it may be elevated in obese PCOS subjects.

In PCOS subjects, 25(OH)D_3_ positively correlated with LEG9, an inflammatory protein significantly upregulated in PCOS women in this study. Of the nine proteins we found upregulated in PCOS, LEG9 showed the most significant association with PCOS in the regression model and ROC analysis, demonstrating 69.0% sensitivity and 72.4% specificity and suggesting its utility as a predictive marker of PCOS.

A positive association of TARC was noted with 1,25(OH)_2_D_3_ in control participants. This could indicate that TARC, an inflammatory chemokine, might be required to maintain homeostasis in healthy participants. Whilst further studies are required to establish the molecular interactions underpinning this observation, it is interesting as 1,25(OH)_2_D_3_ has been suggested to have anti-inflammatory properties in its own right [78] and, hypothetically, may increase in response to increased TARC.

PAPPA, a metalloproteinase, negatively correlated with 1,25(OH)_2_D_3_ in control participants, an association lost in PCOS. Öztürk et al. reported upregulation of PAPPA in lean PCOS women, and thus, it could serve as a clinical indicator of PCOS with CV risk [79]. Our study found a trend for PAPPA upregulation in PCOS, though this did not reach significance. Further investigation with a larger cohort is required to establish the interactions of PAPPA and its associations with 1,25(OH)_2_D_3_ at the molecular level.

Strengths of this study include that the cohort was a well-defined non-obese non-IR Caucasian PCOS population closely matched to controls, allowing comparison of a panel of proteins established as indicative of CV risk without the bias of BMI and IR. Limitations include the small population size; that the study was performed on an exclusively Caucasian population could also be seen as a limitation and would need to be repeated in other ethnic groups to confirm these findings. Further molecular-level analysis is required to validate the potential role of predictive protein candidates such as LEG9 as clinical indicators of PCOS in non-obese individuals.

## 4. Materials and Methods

### 4.1. Study Design

In this exploratory cross-sectional study, plasma CVRP levels were determined in women with PCOS (*n* = 29) and control (*n* = 29) women, all recruited from the Hull IVF clinic [21]. Control and PCOS women were age- and BMI-matched. All procedures accorded with the ethical standards of the Yorkshire and The Humber NRES ethical committee, UK (ethics number 02/03/043), which granted approval for the study. For PCOS diagnosis, Rotterdam consensus criteria were employed: (1) biochemical (free androgen index (FAI) > 4) and clinical (Ferriman–Gallwey score > 8) hyperandrogenaemia (2) amenorrhea or oligomenorrhea and (3) transvaginal ultrasound diagnosis of polycystic ovaries [80]. No other condition/illness was present in the study participants, and all were medication-free (including no over-the-counter medications) for ≥9 months prior to enrolment. Testing to exclude the following endocrine conditions was undertaken: Cushing’s disease, hyperprolactinemia, non-classical 21-hydroxylase deficiency or an androgen-secreting tumor [2]. Demographic and biochemical characteristics of the control and PCOS women are presented in Table 1.

After overnight fasting, waist circumference, height (centimeters) and weight (kilograms) (to calculate body mass index (BMI) using the formula kg/m^2^) were measured per WHO guidelines [81]. Fasting bloods were centrifuged (3500× *g* 15 min), aliquoted and stored (−80 °C). Sex hormone-binding globulin (SHBG), insulin (DPC Immulite 200 analyser, Euro/DPC, Llanberis, UK) and plasma glucose (for calculation of homeostasis model assessment-IR (HOMA-IR)) (Synchron LX20 analyser, Beckman-Coulter, High Wycombe, UK) were measured. FAI was calculated by dividing total testosterone by SHBG × 100. Serum testosterone was determined by isotope-dilution liquid chromatography tandem mass spectrometry (LC-MS/MS) [21], as was vitamin D [82].

Plasma CVRPs were quantified by the Slow Off-rate Modified Aptamer (SOMA)-scan platform [83] as described previously [84,85] (SOMAscan Version 3.1, Somalogic, Boulder, CO, USA). In brief, EDTA plasma samples were treated according to the following steps: (1) Analyte/primer beads-binding SOMAmers (consisting of a synthetic fluorophore-labeled SOMAmer bound via a photocleavable linker to a biotin moiety) underwent equilibration; (2) Analyte/SOMAmer complexes were immobilized using a streptavidin-substituted matrix; (3) UV light was used to cleave, and thus analyte–SOMAmer complexes were released into solution; (4) Subsequent immobilization of analyte–SOMAmer complexes via analyte-borne biotinylation onto a streptavidin matrix; (5) Elution of complexes allowing release of SOMAmers that act as analyte quantification surrogates; (6) Hybridization to SOMAmer-complementary oligonucleotides to enable quantification. Standards were used for calibration as described previously [86]. Standardization and normalization of raw intensities, hybridization, median signal and calibration signal were undertaken [83,86].

SOMAscan Version 3.1 was utilized, targeting the 54 CVRPs, which are detailed in Table 2.

### 4.2. Statistics

This was an exploratory study as there are no studies on the CVRP panel and their relationship to vitamin D; therefore, it was not possible to perform a formal power calculation. Descriptive statistics were used to compute the mean ± SD and the median (IQR) for the continuous variables. The Student *t*-test or Wilcoxon rank sum test was used to assess the statistical differences of the continuous variables across the groups. All tests were two-tailed, and a *p*-value < 0.05 was considered significant.

The quantile-normalized SOMAscan proteomic data were log-transformed before further analysis. Classification of the non-obese PCOS versus control cohorts was initially attempted with hierarchical clustering using Ward clustering and Pearson distances but was unsuccessful. Hence, two sample class comparisons were conducted using linear models for microarray analysis (limma), wherein linear models are combined with t-statistics to find the proteins that were significantly regulated in PCOS. Proteins with a positive or negative fold change greater than 1 with a raw *p*-value less than 0.05 were screened. Correlation analyses between vitamin D and the CVRPs were performed using the Pearson coefficient method. The correlation was considered weak if the correlation coefficient, r, was <0.3, moderate if r was between 0.4 and 0.6 and was considered strong if r was >0.7. The diagnostic value of the individual or the combination of DEGs detected was assessed using univariate logistic regression or multivariable stepwise logistics regression. The area under the curve (AUC) method was used to assess the diagnostic accuracy. All analyses were performed using R Bioconductor packages, SPSS v 2 6.0 and GraphPad Prism version 10.0.0 (San Diego, CA, USA).

### 4.3. Systems Biology Analysis

The list of differentially expressed proteins was used in further downstream analysis. Gene ontology (GO) analysis and the functional enrichment of the dysregulated proteins were conducted using Database for Annotation, Visualization and Integrated Discovery (DAVID) [https://david.ncifcrf.gov/, accessed on 15 March 2024]. Network analysis using the KEGG Charts in the DAVID tool was also performed. The list of dysregulated proteins was subjected to STRING 12.0 database (https://string-db.org/) analysis for generation of the protein–protein interaction network (PPIN).

## 5. Conclusions

Cardiovascular risk proteins, related to inflammation and the immune response, were elevated in women with PCOS who were not obese, and did not have IR or systemic inflammation, and CVRPs correlated with 25(OH)D_3_ levels. This suggests that there is early inflammation inherent in PCOS, and that both PCOS and low vitamin D levels may contribute to inflammation, potentially exacerbating cardiovascular risk.

## Figures and Tables

**Table 1:** Demographics, baseline, hormonal and metabolic parameters of the PCOS subjects and controls (mean ± SD).

	Control (*n* = 29)	PCOS (*n* = 29)	*p*-Value
Age (years)	32.5 ± 4.1	31 ± 6.4	0.14
BMI (kg/m^2^)	24.8 ± 1.1	25.9 ± 1.8	0.56
Fasting glucose (nmol/L)	4.9 ± 0.4	4.7 ± 0.8	0.06
HbA1C (mmol/mol)	30.9 ± 6.5	31.8 ± 3.0	0.9
HOMA-IR	1.8 ± 1.0	1.9 ± 1.6	0.97
SHBG (nmol/L)	104.2 ± 80.3	71.7 ± 62.2	0.01
Free androgen index (FAI)	1.3 ± 0.5	4.1 ± 2.9 ± 4.08	0.0001
CRP (mg L^−1^)	2.34 ± 2.34	2.77 ± 2.57	0.43
AMH (ng/mL)	24.3 ± 13.1	57.2 ± 14.2	0.0001
25(OH)D_3_ (laboratory reference range 20–40 ng/mL)	23.77 (13.72–33.24)	21.80 (15.63–30.46)	0.86
1,25(OH)_2_D_3_ (laboratory reference range 0.02–0.08 ng/mL)	0.04 (0.03–0.05)	0.05 (0.035–0.06)	0.53

BMI—Body Mass Index; HbA1c—glycated hemoglobin; HOMA-IR—Homeostasis model of assessment—IR; CRP—C-reactive protein; SHBG—sex hormone-binding globulin; AMH—anti-Müllerian hormone.

**Table 2:** Cardiovascular risk proteins (CVRPs) analyzed using SOMAscan.

Genes	Target Protein
*BMP-6*	Bone morphogenetic protein 6
*SLAF7*	SLAM family member 7
*ATS13*	A disintegrin and metalloproteinase with thrombospondin motifs 13
*Angiopoietin-1*	Angiopoietin-1
*Adrenomedullin*	Adrenomedullin
*ATS13*	A disintegrin and metalloproteinase with thrombospondin motifs 13
*SRCN1*	Proto-oncogene tyrosine-protein kinase Src
*IL-6*	Interleukin-6
*TRAIL R1*	Tumor necrosis factor receptor superfamily member 10A
*IDUA*	Alpha-L-iduronidase
*RANK*	Tumor necrosis factor receptor superfamily member 11A
*TRAIL R2*	Tumor necrosis factor receptor superfamily member 10B
*Marapsin*	Serine protease 27
*sTie-2*	Angiopoietin-1 receptor, soluble
*TF*	Tissue Factor
*PDGF Rb*	Platelet-derived growth factor receptor beta
*IL-27*	Interleukin-27
*Gro-a (CXCL1)*	Growth-regulated alpha protein
*PIGR*	Polymeric immunoglobulin receptor
*sRAGE*	Advanced glycosylation end product-specific receptor, soluble
*Mn SOD*	Superoxide dismutase [Mn], mitochondrial
*HGH*	Somatotropin
*FST*	Follistatin
*IL-1Ra*	Interleukin-1 receptor antagonist protein
*PIGF*	Placenta growth factor
*BOC*	Brother of CDO
*LEG9*	Galectin-9
*IL-18 Ra*	Interleukin-18 receptor 1
*FCG2B*	Low-affinity immunoglobulin gamma Fc region receptor II-b
*Tpo*	Thrombopoietin
*MIP-1a*	C-C motif chemokine 3
*SLAF5*	SLAM family member 5
*PAPPA*	Pappalysin-1
*Renin*	Renin
*TSP2*	Thrombospondin-2
*Lymphotactin*	Lymphotactin
*IL-16*	Interleukin-16
*TARC*	C-C motif chemokine 17
*MMP-7*	Matrilysin
*Bone proteoglycan II*	Decorin
*DKK1*	Dickkopf-related protein 1
*ART*	Agouti-related protein
*HB-EGF*	Heparin-binding EGF-like growth factor
*GDF2*	Growth/differentiation factor 2
*MMP-12*	Macrophage metalloelastase
*ACE2*	Angiotensin-converting enzyme 2
*PD-L2*	Programmed cell death 1 ligand 2
*TACI*	Tumor necrosis factor receptor superfamily member 13B
*Leptin*	Leptin
*sCD4*	T-cell surface glycoprotein CD4
*IgE*	Immunoglobulin E
*FGF23*	Fibroblast growth factor 23
*BNP-32*	Brain natriuretic peptide 32
*IL-2 sRa*	Interleukin-2 receptor subunit alpha

**Table 3:** Cardiovascular risk biomarkers (CVRPs) that differed between non-obese weight-matched women with and without polycystic ovary syndrome (PCOS).

CVRPs	logFC	Av Expression	T	*p* Value
LEG9	0.24	9.95	2.4	0.02
BOC	0.50	10.33	2.4	0.02
MIP-1a	0.27	8.87	2.4	0.02
IL-18 Ra	0.36	12.98	2.3	0.03
Tpo	0.29	6.92	2.2	0.03
IL-1Ra	0.39	11.93	2.2	0.03
PD-L2	0.26	11.55	2.2	0.03
FCG2B	0.45	10.64	2.1	0.04
HGH	0.24	7.94	2.0	0.04

LEG9 = Galectin-9; BOC = Brother of CDO; MIP-1a = C-C motif chemokine 3; IL-18 Ra = Interleukin-18 receptor 1; Tpo = Thrombopoietin; IL-1Ra = Interleukin-1 receptor antagonist protein; PD-L2 = Programmed cell death 1 ligand 2; FCG2B = Low-affinity immunoglobulin gamma Fc region receptor II-b; HGH = human growth hormone.

**Table 4:** Significant enrichment terms associated with biological process and Kyoto Encyclopedia of Genes and Genomes DAVID: Database for Annotation, Visualization and Integrated Discovery (KEGG) pathway carried out using the DAVID online tool for the upregulated proteins in the PCOS non-obese cohort when compared to the healthy non-obese cohort as identified by SOMAscan assay.

GO Terms	Count	*p*-Value	Benjamini
**Biological Processes—Upregulated genes**			
GO:0006954—inflammatory response	4	5.2 × 10^−4^	5.5 × 10^−2^
GO:0006955—immune response	4	8.6 × 10^−4^	5.5 × 10^−2^
GO:0032760—positive regulation of TNF production	3	9.0 × 10^−4^	5.5 × 10^−2^
**Pathway enrichment—Upregulated genes**			
KEGG_Pathway term: cytokine–cytokine receptor interaction	5	4.4 × 10^−5^	1.1 × 10^−3^

**Table 5:** Cardiovascular risk biomarkers that correlated with 25(OH)D_3_ and 1,25(OH)_2_D_3_ in non-obese weight-matched women with and without PCOS. 25(OH)D_3_ was positively correlated with one CVRP and was negatively correlated with four CVRPs in the PCOS cohort.

PCOS		
	25(OH)D_3_	
	r	*p*
IDUA	−0.570	0.01
LEG9	0.518	0.02
Angiopoietin-1	−0.500	0.02
DKK1	−0.458	0.04
Gro-a (CXCL1)	−0.437	0.04
**CONTROL**		
	**25(OH)D_3_**	
	**r**	***p***
MMP7	0.41	0.03
	**1,25(OH)_2_D_3_**	
TARC	0.68	0.001
PAPPA	−0.48	0.03

## Data Availability

The raw data supporting the conclusions of this article will be made available by the authors upon request.
